# Biome-specific distribution of Ni-containing carbon monoxide dehydrogenases

**DOI:** 10.1007/s00792-022-01259-y

**Published:** 2022-01-20

**Authors:** Masao Inoue, Kimiho Omae, Issei Nakamoto, Ryoma Kamikawa, Takashi Yoshida, Yoshihiko Sako

**Affiliations:** 1 Graduate School of Agriculture, Kyoto University, Kitashirakawa Oiwake-cho, Sakyo-ku, Kyoto, 606-8502 Japan; 2 R-GIRO, Ritsumeikan University, 1-1-1 Nojihigashi, Kusatsu, Shiga 525-8577 Japan; 3 College of Life Sciences, Ritsumeikan University, 1-1-1 Nojihigashi, Kusatsu, Shiga 525-8577 Japan; 4 Department of Integrated Biosciences, Graduate School of Frontier Science, The University of Tokyo, 5-1-5 Kashiwanoha, Kashiwa, Chiba 277-8561 Japan

**Keywords:** Carbon monoxide, Microbiome, Metagenome, Carbon monoxide dehydrogenase, Phylogeny, Protein evolution

## Abstract

Ni-containing carbon monoxide dehydrogenase (Ni-CODH) plays an important role in the CO/CO_2_-based carbon and energy metabolism of microbiomes. Ni-CODH is classified into distinct phylogenetic clades, A–G, with possibly distinct cellular roles. However, the types of Ni-CODH clade used by organisms in different microbiomes are unknown. Here, we conducted a metagenomic survey of a protein database to determine the relationship between the phylogeny and biome distribution of Ni-CODHs. Clustering and phylogenetic analyses showed that the metagenome assembly-derived Ni-CODH sequences were distributed in ~ 60% Ni-CODH clusters and in all Ni-CODH clades. We also identified a novel Ni-CODH clade, clade H. Biome mapping on the Ni-CODH phylogenetic tree revealed that Ni-CODHs of almost all the clades were found in natural aquatic environmental and engineered samples, whereas those of specific subclades were found only in host-associated samples. These results are comparable with our finding that the diversity in the phylum-level taxonomy of host-associated Ni-CODH owners is statistically different from those of the other biomes. Our findings suggest that while Ni-CODH is a ubiquitous enzyme produced across diverse microbiomes, its distribution in each clade is biased and mainly affected by the distinct composition of microbiomes.

**Supplementary Information:**

The online version contains supplementary material available at 10.1007/s00792-022-01259-y.

## Introduction

Ni-containing carbon-monoxide dehydrogenase (Ni-CODH) is a primordial microbial enzyme that catalyzes the reversible conversion between CO_2_ and CO (CO_2_ + 2H^+^  + 2e^−^ ⇔ CO + H_2_O), a key reaction for carbon fixation and energy conservation (Oelgeschläger and Rother 2008; Sokolova et al. 2009; Nitschke and Russell 2013; Can et al. 2014; Adam et al. 2018; Inoue et al. 2019a; Schoelmerich and Müller 2019). CO_2_ reduction by the Ni-CODH/CO-methylating acetyl-CoA synthase complex constitutes the carbonyl branch of the Wood–Ljungdahl pathway (WLP) in acetogens and methanogens (Doukov et al. 2002; Gong et al. 2008; Nitschke and Russell 2013; Can et al. 2014; Schuchmann and Müller 2014). The WLP is widely distributed in bacteria and archaea, and was also present in the last universal common ancestor (Adam et al. 2018). The WLP also uses CO as a direct input and is the major pathway for carbon cycling in both anabolic and catabolic manners for carboxydotrophy (Tan et al. 2005). In a catabolic manner, Ni-CODH is a part of the respiratory module via CO oxidation in anaerobic carboxydotrophs, where electrons from CO are finally transferred to various terminal electron acceptors (e.g., H^+^, Fe^3+^, fumarate, sulfur oxides, and nitrogen oxides) via the corresponding respiratory enzymes (Oelgeschläger and Rother 2008; Sokolova et al. 2009; Fukuyama et al. 2020). Because of the low redox potential of CO (*E*^0′^ =  − 520 mV), the coupling of CO oxidation and H^+^ reduction is undertaken by the Ni-CODH/electron carrier polyferredoxin CooF/energy converting hydrogenase (ECH) respiratory complex, which is an ancient respiratory module (Soboh et al. 2002; Singer et al. 2006; Schoelmerich and Müller 2019). In addition, Ni-CODH associated with flavin adenine dinucleotide-dependent NAD(P) oxidoreductase (FNOR) and CooF is considered to enable NAD(P)H-mediated CO-driven respiration (Whitham et al. 2015; Geelhoed et al. 2016; Slobodkin et al. 2019).

Ni-CODHs are phylogenetically diverse enzymes that are classified into seven distinct clades from A to G (Inoue et al. 2019a). In addition, a small Ni-CODH-like protein called mini-CooS has the divergent amino acid sequence but is found in the carboxydotroph *Thermosinus carboxydivorans* with a complete motif for metal cluster formation (Techtmann et al. 2012). The tertiary structures and catalytic activities of Ni-CODHs in clades A, E, and F have been characterized in model carboxydotrophs such as *Methanosarcina barkeri*, *Carboxydothermus hydrogenoformans*, *Rhodospirillum rubrum*, *Moorella thermoacetica*, and *Desulfovibrio vulgaris* (Dobbek et al. 2001; Drennan et al. 2001; Doukov et al. 2002; Gong et al. 2008; Benvenuti et al. 2020; Wittenborn et al. 2020), whereas those in other clades have not been determined yet. However, the possible involvement of clades C and D in CO oxidation has been suggested (Rother et al. 2007; Whitham et al. 2015; Liew et al. 2016). The horizontal gene transfer and loss of Ni-CODH genes have led to the punctate distribution of the A–G clades in the prokaryotic tree of life; hence, all of them are found in both bacteria and archaea (Techtmann et al. 2012; Inoue et al. 2019a).

The Ni-CODH genes often form gene clusters with functionally associated genes in microbial genomes (Techtmann et al. 2012; Inoue et al. 2019a), such as the genes for metallochaperones CooC and CooT as maturation factors, or CO-responsive transcription factors in addition to the WLP, ECH, FNOR, or CooF. This feature essentially provides clues for predicting novel Ni-CODH-associated genes such as the functionally unknown NitT/TauT-family ABC transporters (Inoue et al. 2019a). The relationship between these Ni-CODH-associated genes and the phylogenetic clades of Ni-CODH has also been established. The WLP genes are associated with clades A, E, and F; the CooF/ECH genes are associated with clades E and F; the CooF/FNOR genes are associated with clades C–G; and the ABC transporter genes are associated with clades B and D–F. Some anaerobic carboxydotrophs have multiple gene clusters, of which Ni-CODHs belong to different phylogenetic clades. The expression of these Ni-CODH gene clusters is regulated differently in the presence or absence of CO in some carboxydotrophs (Fukuyama et al. 2019; Inoue et al. 2020a).

Interestingly, carboxydotrophs can grow even under one atmosphere pressure of CO (Robb and Techtmann 2018), despite it being toxic for many microbes (Wareham et al. 2018). Furthermore, many carboxydotrophs have been isolated from CO-emitting volcanic environments (Fukuyama et al. 2020). However, some carboxydotrophs have also been isolated from samples not related to CO-emitting environments, suggesting their ubiquitous distribution (Inoue et al. 2019b, 2020b). Ni-CODH genes have been identified in metagenome-assembled genomes (MAGs) from various environmental samples (Evans et al. 2015; Adam et al. 2018; Inoue et al. 2019a; Mu et al. 2020). These evidences have helped establish an enigmatic relationship between the physiology and ecology of carboxydotrophs. A conceptual model represents the role of carboxydotrophy in microbiomes, that is removals of locally accumulated biogenic CO from microbial end products (Robb and Techtmann 2018). Several molecular ecological studies have reported Ni-CODH sequences from the biomes of the termite hindgut, deep sea sediments, and hot springs, which unveiled the relationships between the biomes and the phylogenetic clades of Ni-CODHs (Matson et al. 2011; Hoshino and Inagaki 2017; Omae et al. 2021). The Ni-CODH sequences from the termite gut biome were different from those of the deep-sea sediment samples even when the same primers were used, implying that different biomes accommodate different phylogenetic clades or functions of Ni-CODHs (Matson et al. 2011; Hoshino and Inagaki 2017). However, these studies used PCR-based DNA metabarcoding, where primer specificity might cause biased amplification, thereby hampering the sequencing results. Therefore, PCR-free, genome-based or metagenome-based evaluation of Ni-CODH distribution in different biomes is required to gain deeper insight into the ecological roles of Ni-CODHs and the carboxydotrophs producing them.

To this end, we performed a comprehensive biome tagging over the entire Ni-CODH phylogenetic tree using the state-of-the-art metagenomics protein sequence database, MGnify. It contains over 1,100,000,000 protein sequences in over 12,000 metagenomic assemblies from various biome samples that are mainly categorized into “engineered,” “environmental (aquatic),” or “host-associated” (Mitchell et al. 2020). To construct a phylogenetically unbiased dataset, we adopted a sequence clustering approach using approximately 27,000 Ni-CODH sequences derived from MGnify and over 3,000 reference Ni-CODH sequences from RefSeq/GenBank with genomic information. We found a new clade H of Ni-CODH, in addition to the previously known clades A–G. We also demonstrated that phylogenetically diverse Ni-CODH clusters in clades A–H were found in both “environmental (aquatic)” and “engineered” samples, whereas specific groups in the clades B–E were found mainly in “host-associated” samples, showing a broad but biased distribution of Ni-CODHs. These distributions of Ni-CODHs were related to the taxonomy of Ni-CODH-harboring microbes. Our findings suggest that the biomes could affect the phylogenetic diversity of Ni-CODHs. Also, while Ni-CODH is a ubiquitous enzyme across the diverse microbiomes, its distribution in each clade is biased and mainly affected by the distinct microbiome composition.

## Materials and methods

### Construction of Ni-CODH protein datasets

The amino acid sequence retrieval for Ni-CODHs was performed using the DIAMOND BLASTp search version 0.9.29 (Buchfink et al. 2014) with an e-value cutoff of < 10^−3^ from the MGnify protein database (Mitchell et al. 2020) (May, 2019; containing ~ 1,100,000,000 protein sequences from 12,489 assemblies) and the National Center for Biotechnology Information (NCBI) RefSeq/GenBank non-redundant protein sequence database (Sayers et al. 2020) (February, 2020; containing ~ 260,000,000 protein sequences). The query sequences for sequence retrieval are as follows: WP_011305243.1, WP_026514536.1, WP_039226206.1, WP_011342982.1, WP_012571978.1, WP_011343033.1, OGP75751.1, and WP_007288589.1 for clades A to G Ni-CODHs (Inoue et al. 2019a) and mini-CooS (Techtmann et al. 2012), respectively. For obtaining Ni-CODH representative sequences from MGnify and RefSeq/GenBank, short-length hits (amino acid length < 400) were excluded, and then the sequences with deletions or mutations in the conserved residues for the metal cluster and the active site were filtered out by verifying the multiple sequence alignment prepared using the MAFFT version 7.471 program with the E-INS-i method (Katoh and Standley 2013). The criteria for sequence filtering were the same as described previously (Inoue et al. 2019a) with minor modifications: no deletions in the D-, B-, C-clusters, and the catalytic residues, and no mutation in the four Cys residues forming the B-cluster and the three C-terminal Cys residues forming the C-cluster. The excluded sequences in the MGnify dataset with an amino acid length of 100 or more in the above steps were only used for the following clustering step to increase the depth of biome tagging (see below). In addition, the Ni-CODH-encoding genomes were retrieved from the NCBI assembly database (Sayers et al. 2020) (February, 2020; covering ~ 240,000 prokaryotic genomes from isolates and MAGs) by searching for protein accession numbers throughout "feature tables" of all prokaryotic genome assemblies. The RefSeq/GenBank sequences without genomic information were excluded. The workflow of the data retrieval and analysis pipeline is summarized in Fig. S1.

### Sequence clustering

Sequence clustering was performed using the UCLUST algorithm in USEARCH version 11.0.667 (Edgar 2010) at 90% sequence identity with the following order of priority: (1) the RefSeq/GenBank Ni-CODH representatives, (2) the MGnify Ni-CODH representatives, and (3) the MGnify Ni-CODH-like sequences (≥ 100 aa) excluded in the filtering step, all of which were sorted in the descending order of length. The RefSeq/GenBank and MGnify representatives were selected accordingly as centroids for the clusters.

### Construction of phylogenetic tree

The 2462 Ni-CODH centroid sequences of the clusters were aligned using the MAFFT program with the E-INS-I method (Katoh and Standley 2013). Ambiguously aligned sites were subsequently trimmed using the trimAl version 1.4.1 program with the "automated1" mode (Capella-Gutiérrez et al. 2009). The trimmed alignment for phylogenetic analysis contained 440 amino acid sites that were used for the maximum likelihood phylogenetic tree reconstruction using IQTREE version 2.0.3 (Minh et al. 2020) with the LG + R10 model assigned by ModelFinder (Kalyaanamoorthy et al. 2017). The reliability of the tree topology was evaluated by UFBoot ultrafast bootstrapping in IQTREE based on 1000 resamplings (Hoang et al. 2018). Phylogenetic classification of Ni-CODHs was performed as described previously (Inoue et al. 2019a). The trees were visualized using the R package "ggtree" version 2.2.4 (Yu et al. 2017).

### Classification of biome and genome information

Biome annotation for the clustered MGnify Ni-CODH sequences was performed according to the MGnify biome classification (Mitchell et al. 2020). To remove detailed lower rank subcategories, we used “engineered,” “environmental (aquatic),” and “host-associated” as the major categories. These categories were further divided into the following subcategories: “engineered” into “biogas plant,” “bioreactor,” “bioremediation,” “food production,” “solid waste,” and “waste water”; “environmental (aquatic)” into “aquaculture,” “estuary,” “freshwater,” “marine,” and “thermal springs”; “host-associated” into “human digestive system,” “human skin,” “mammals digestive system,” and “mammals respiratory systems”. Note that “human skin” was excluded from the data analysis because only one sequence was classified into this subcategory.

Genome-based taxonomies for the genomes encoding the RefSeq/GenBank Ni-CODHs were annotated using GTDBTk version 1.3.0 with the GTDB database release R95 (Parks et al. 2020; Chaumeil et al. 2020) based on fastANI comparison (Jain et al. 2018) and phylogenetic placement (Matsen et al. 2010). The genomic contexts of the RefSeq/GenBank Ni-CODH genes were assigned as described previously with minor modifications (Omae et al. 2019). Fifteen coding sequences upstream and downstream of the Ni-CODH gene locus were annotated by the clusters of orthologous groups of proteins (COGs) (Galperin et al. 2015) via the RPS-BLAST search with an e-value cutoff of < 10^−6^ using the NCBI Conserved Domain Database (Marchler-Bauer et al. 2017). Neighboring gene-associated functions of the Ni-CODH genes were categorized by the previously described COGs (Inoue et al. 2019a) found in each genomic context: WLP (COG1614), CooF (COG0437 or COG1142), FNOR (COG1251), ECH (COG3260 and COG3261), ABC transporter (COG0600, COG1116, and COG0715), and metallochaperone (COG3640 or COG1532).

### Data analysis

Similarity scores based on the Dice similarity coefficients were calculated from the co-occurrence patterns of biomes, Ni-CODH clades, genome-based taxonomies, and neighboring gene-associated functions within the Ni-CODH sequence clusters using the R package "proxy" version 0.4–19. The composition of Ni-CODH clades, genome-based taxonomies, and neighboring gene-associated functions in each biome were compared by principal component analysis using R and visualized by the R package "ggplot2" version 3.3.0.

## Results

### Numerous Ni-CODHs were identified from metagenomic contigs

First, we retrieved 5430 and 3164 Ni-CODH sequences from the MGnify and RefSeq/GenBank databases, respectively, as representative sequences, which had conserved sequence motifs (Inoue et al. 2019a) and sufficient lengths for phylogenetic analysis (Fig. S1). Next, we performed clustering at 90% amino acid sequence identity using these MGnify and RefSeq/GenBank representative sequences and unfiltered MGnify sequences including short length hits (≥ 100 aa), in which the representatives were selected as centroids. Thus, 2462 Ni-CODH clusters were generated from the 30,112 Ni-CODH sequences (Fig. S2 and Table S1). The clustered 26,948 MGnify sequences were derived from 6348 sequence assemblies/runs with 4817 samples corresponding to approximately half of all assemblies from the MGnify database in the range of 0.001 to 8 per Mbp (Fig. S3), whereas the 3164 RefSeq/GenBank sequences were encoded by 8946 gene locus tags in 5301 genomes from 66 phyla in the GTDB genome-based taxonomy. The clusters consisted of 615 clusters with both MGnify and RefSeq/GenBank sequences, 809 clusters with only MGnify sequences, and 1038 clusters with only RefSeq/GenBank sequences, indicating that the MGnify dataset expanded Ni-CODH sequence space by ~ 30% at the level of 90% sequence identity.

The clustering criterion (90% amino acid sequence identity) used in this study was selected on the basis of the compression rate and the number of total sequences (Fig. S2). If the clustering criterion is precisely selected, each cluster would be composed of sequences from closely related species or single species, except for some horizontally transferred cases. In our study, ~ 94% and ~ 78% of the Ni-CODH clusters consisted of sequences from a single genus and species, respectively, even though horizontal gene transfers have been reported (Techtmann et al. 2012; Inoue et al. 2019a). The genome-based taxonomy annotation supported the clustering criterion of 90%.

### Metagenomic contig-derived Ni-CODHs were phylogenetically diverse

**Fig. 1:**
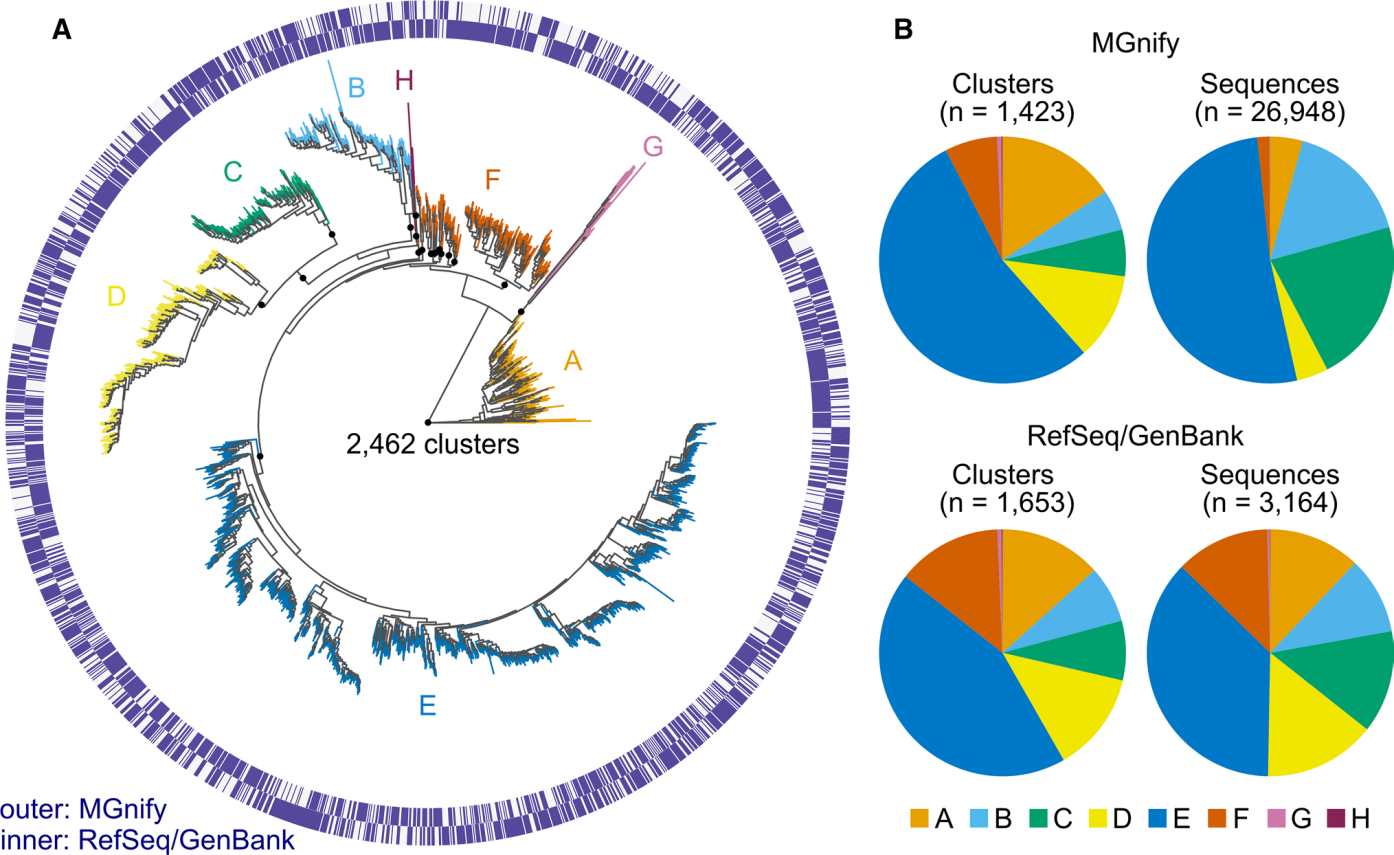
Distribution of MGnify and RefSeq/GenBank sequences on Ni-CODH phylogeny. **A** A phylogenetic of Ni-CODH clusters mapped with navy lines along the two circles showing the presence of MGnify (outer) and RefSeq/GenBank (inner) sequences in each cluster. An unrooted phylogenetic tree was constructed using an alignment of centroid sequences from the 2462 Ni-CODH clusters on 90% amino acid sequence identity. The major clades A to H are highlighted by different colors as follows: yellow orange, clade A; light blue, clade B; green, clade C; yellow, clade D; blue clade E; red orange, clade F; pale magenta, clade G; and magenta, clade H. The black circles on the branches separating the major clades or subclades indicate their bootstrap value of > 0.95 support. **B** Pie charts for clade compositions in Ni-CODH clusters and sequences with MGnify and RefSeq/GenBank

To reveal the phylogenetic diversity of the MGnify Ni-CODHs, we constructed phylogenetic tree using the centroid sequences of the 2462 Ni-CODH clusters, and then assigned the previously reported seven major clades A–G (Inoue et al. 2019a) and mini-CooS (Fig. 1A). Clades A–E showed monophyletic topology, whereas clade F was reconstructed paraphyletically, as reported in a previous large-scale analysis using IQ-TREE (Adam et al. 2018). Clade G and mini-CooS formed a monophyletic clade that branched between clade A and the other clades as described previously (Techtmann et al. 2012; Inoue et al. 2019a). Therefore, mini-CooS was also considered a member of clade G. We also found the novel phylogenetic clades (collectively referred to as clade H) including the two RefSeq/GenBank sequences, which were previously unidentified since they had been excluded because of differences in the selection criteria. Both the MGnify and RefSeq/GenBank sequences were found in all clades and broadly distributed in the Ni-CODH phylogeny (Fig. 1A). Although the fraction of Ni-CODH clusters containing the MGnify sequences in clade F (7%) was twofold lower than the fraction of Ni-CODH clusters containing RefSeq/GenBank sequences (14%) (Fig. 1B), the overall clade composition was similar between MGnify and RefSeq/GenBank. Thus, our dataset was sufficient to tag the biome and genomic information on Ni-CODH phylogeny. Moreover, it is noteworthy that our clustering approach eliminated the clade composition bias in the MGnify sequences (Fig. 1B), providing a relatively unbiased biome distribution.

### Biome distribution of Ni-CODHs was different depending on their phylogeny

**Fig. 2:**
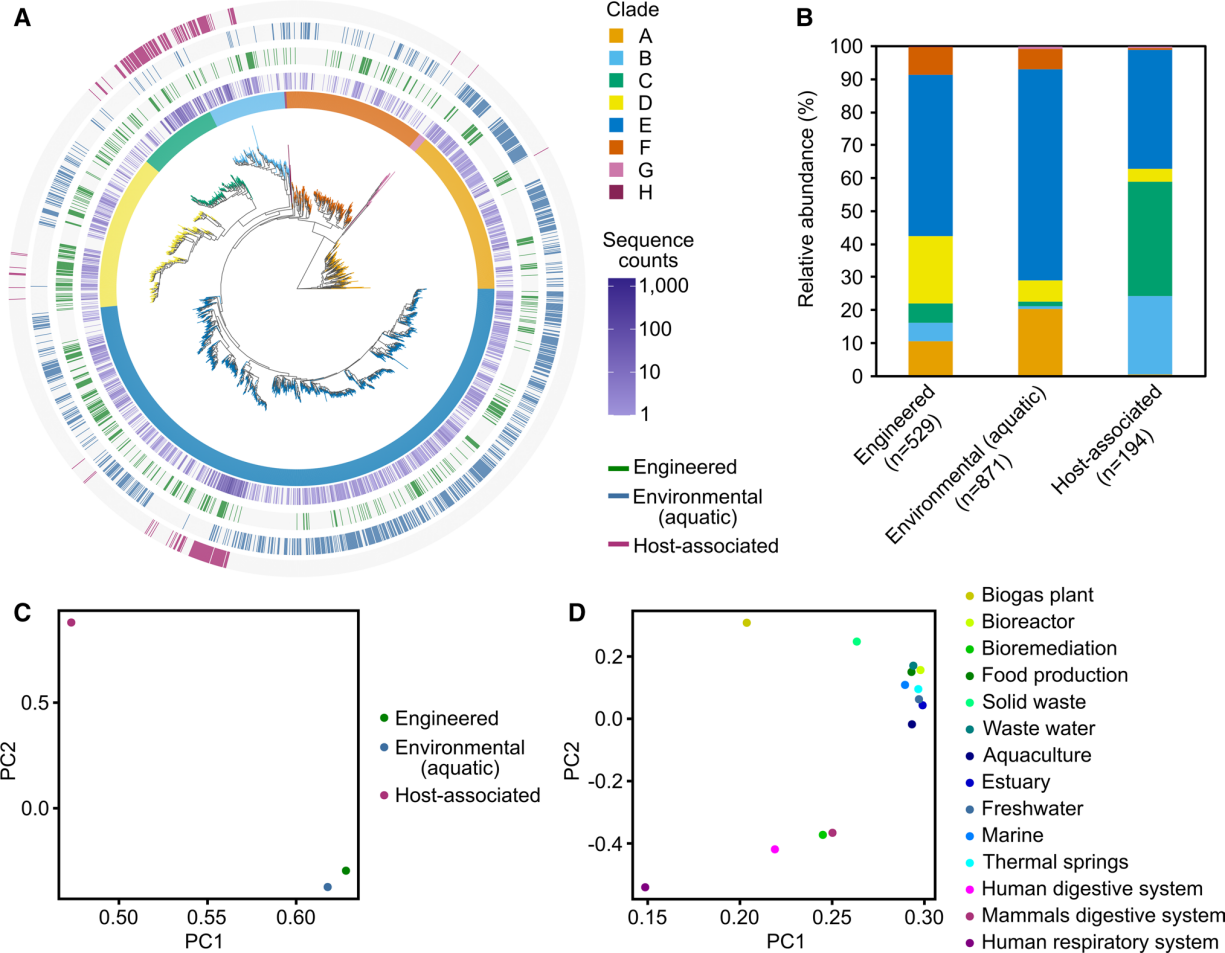
Biome mapping to whole Ni-CODH phylogeny. **A** A phylogenetic tree of Ni-CODH clusters as shown in Fig. 1A mapped with three major biomes. The innermost ring represents clades of Ni-CODH clusters by colors as shown in Fig. 1A. The second inner ring indicates relative abundance of MGnify Ni-CODH sequences in each cluster by navy lines with color gradation. The outer three rings represent the presence of MGnify Ni-CODH sequences from three major biomes in the following order from inner to outer: green lines, "engineered"; blue lines, "environmental (aquatic)"; and magenta lines, "host-associated". **B**, **C** Clade compositions of Ni-CODH clusters (**B**) and principal component analysis (**C**) in the three major biome categories. **D** Principal component analysis of clade compositions of Ni-CODH clusters in the fourteen biome subcategories related to Fig. S4

The Ni-CODH clusters containing the MGnify sequences were assigned by biome information to demonstrate the relationships between their phylogeny and biome distribution (Fig. 2A). The RefSeq/GenBank representative sequences contained MAG-derived sequences; nevertheless, biome tagging was performed using only MGnify datasets to unify criteria for biome classification. The clustered 26,948 MGnify Ni-CODH sequences were assigned to the three major biome categories containing 3796 “engineered,” 2967 “environmental (aquatic),” and 20,228 “host-associated” sequences (Table S1), indicating “environmental (aquatic)” and “host-associated” sequences as the least and the most abundant, respectively, in the current dataset. After clustering, a total of 1424 Ni-CODH clusters (~ 60% of all Ni-CODH clusters) were classified into one or more of the three biome categories: 529, 871, and 194 clusters were classified into “engineered,” “environmental (aquatic),” and “host-associated,” respectively (Fig. 2A and Table S1). These data indicated that the “host-associated” Ni-CODHs showed considerably lower diversity in amino acid sequences in spite of the high sequence counts as indicated above, whereas the most diverse Ni-CODHs were found in the smallest sequence space of the “environmental (aquatic)” category.

As shown in Fig. 2A, Ni-CODHs from “environmental (aquatic)” and “engineered” samples were phylogenetically diverse, whereas Ni-CODHs from “host-associated” samples were noticeably restricted to some groups, especially clades B, C, and E, which included 92% of total “host-associated” Ni-CODH clusters. Principal component analysis suggested that the composition of the Ni-CODH clades in the “host-associated” samples was different from the composition of those in the other samples (Fig. 2B, C). The Dice similarity coefficient value determined from co-occurrence in the sequence clusters between “environmental (aquatic)” and “host-associated” (0.046) samples was also three to fourfold lower than the value between “engineered” and “host-associated” (0.14) samples and that between “environmental (aquatic)” and “engineered” (0.18) samples, respectively. These data indicated that the “environmental (aquatic)” and “host-associated” samples had phylogenetically distinct Ni-CODH sequences. The similarity values of the “engineered” samples in combination with the other samples were comparable. Despite the difference in clade compositions, the Ni-CODHs from the “engineered” samples exhibited co-occurrence at the same level with those from the “host-associated” samples as well as "environmental (aquatic)" samples in the Ni-CODH clusters.

The subcategorized biomes (see “Materials and methods”) mapped on the phylogenetic tree exhibited similar distribution patterns among the three major biomes (Fig. S4). Co-occurrence analysis of Ni-CODH clusters showed that the mean value of the Dice similarity coefficients of the three self-combinations within each major category (0.12) was sixfold higher than that of the other three combinations (0.020) (*P* = 0.0016 by Welch’s *t* test). The compositions of the Ni-CODH clades in the subcategorized biomes were similar within each major category, except for the “engineered” category (Fig. 2D). In the “engineered” category, the composition of Ni-CODH clades in “bioremediation” was similar to that of the “host-associated” samples. Similarly, the Dice similarity coefficient value between “bioremediation” and “human digestive system” (0.24) was eightfold higher than the mean value of the all combinations between “engineered” and “host-associated” biomes (*P* = 0.00054 by Grubbs–Smirnov test). Overall, with the few exceptions, the results using subcategorized biomes were similar to those using the three major categories, indicating that the distribution patterns of the distinct clades of the Ni-CODHs were restricted by the major biome categories.

### Phylum-level taxonomies rather than associated protein functions affected biome distribution of Ni-CODHs

Finally, to gain insight into the factors underpinning the restricted Ni-CODH distribution, we analyzed the relationships between biome distribution and genomic information of Ni-CODHs. In particular, we focused on taxonomy of the Ni-CODH-bearing prokaryotes and function of genes located near the Ni-CODH genes, possibly as gene clusters, since these two appeared to be related to the Ni-CODH phylogeny (Inoue et al. 2019a). Genomic information was mapped onto the phylogenetic tree of Ni-CODHs and co-occurrence analysis of biome and genomic information was performed using the 615 Ni-CODH clusters with both MGnify and RefSeq/GenBank sequences (Fig. S5).

**Fig. 3:**
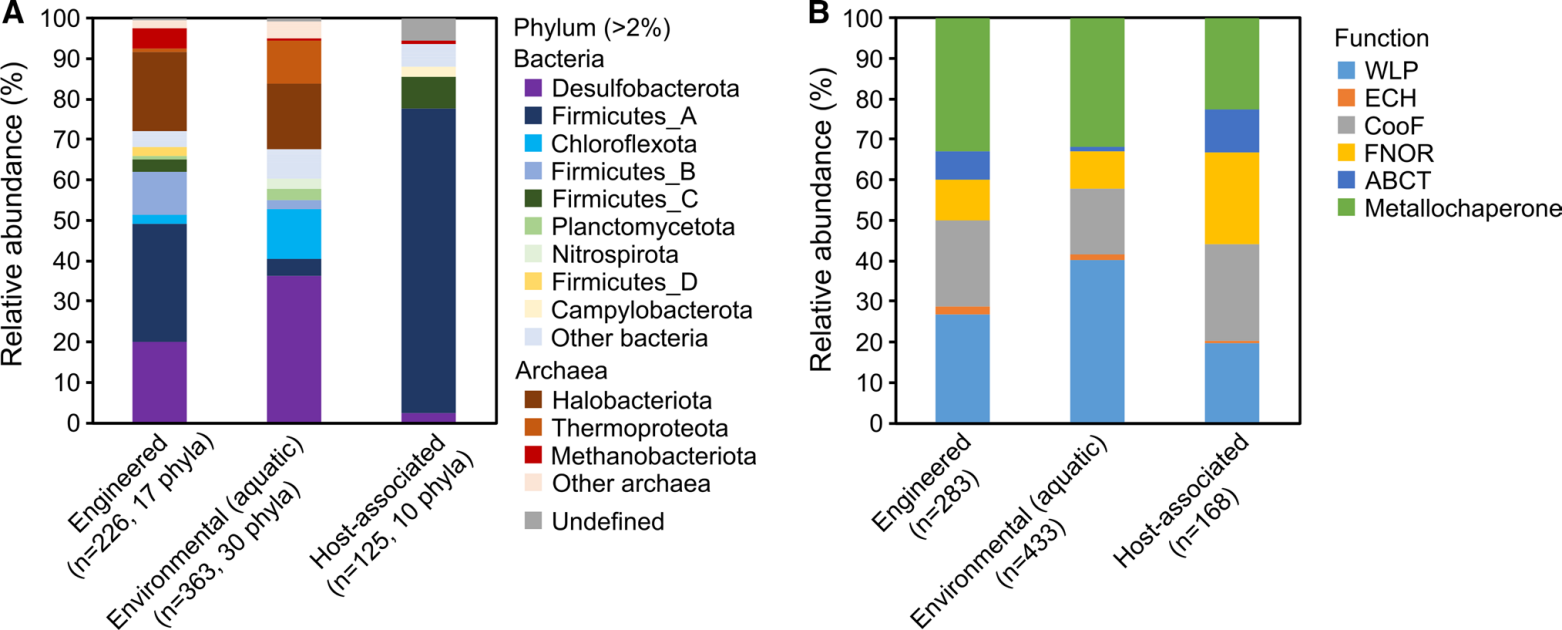
Compositions of phylum-level taxonomies (**A**) and neighboring gene-associated functions (**B**) in three major biome categories annotated by co-occurrence of biome and genomic information

Composition profiles of phylum-level taxonomies of the Ni-CODH owners were different among the three major biomes, which were characterized by major phyla (> 10% relative abundance of clusters) as follows (Fig. 3A): Firmicutes_A, Desulfobacterota, Halobacteriota, and Firmicutes_B in “engineered”; Desulfobacterota, Halobacteriota, Chloroflexota, and Thermoproteota in “environmental (aquatic)”; and Firmicutes_A in “host-associated” samples. The Shannon diversity indices by phylum-level taxonomy of “engineered,” “environmental (aquatic),” and “host-associated” samples were 0.88, 0.95, and 0.46, respectively, indicating the lowest taxonomic diversity of the “host-associated” Ni-CODHs. In fact, ~ 75% of the “host-associated” Ni-CODH clusters were derived from the phylum Firmicutes_A, whereas the remaining 25% were derived from only nine phyla. The order Lachnospirales in Firmicutes_A was a major owner of the “host-associated” Ni-CODH clusters covering 64% of the Ni-CODH clusters from Firmicutes_A in “host-associated” samples, but only 7% of those in “environmental (aquatic)” and “engineered” samples indicating considerable differences even in the lower taxonomic levels, orders, among biomes. These taxonomic differences likely restricted the distribution of the Ni-CODH clades in each biome.

Conversely, the composition patterns of neighboring gene-functions (i.e., WLP, CooF, FNOR, ECH, ABC transporter, and metallochaperone) were similar among the three major biome categories, although slight differences were observed (Fig. 3B). The fraction of the WLP-associated Ni-CODH clusters was relatively more abundant in “environmental (aquatic)” samples while those of the CooF- and FNOR-associated Ni-CODH clusters were higher in “host-associated” samples. A few ECH-related Ni-CODH clusters were detected throughout the three major biomes as described previously (Omae et al. 2019; Fukuyama et al. 2020). These results suggested that functional requirements of Ni-CODHs remain similar among different biomes.

## Discussion

Ni-CODH is a key enzyme for energy conservation and carbon fixation with CO, a mysterious but ubiquitous microbial metabolite (Oelgeschläger and Rother 2008; Nitschke and Russell 2013; Robb and Techtmann 2018). In the present study, we focused on the molecular ecology of Ni-CODHs, especially the relationship between phylogenetic diversity and biome distribution of the enzyme.

The genome-based datasets presented here overcame the limitations of the previous studies using PCR-based methods (Matson et al. 2011; Hoshino and Inagaki 2017; Omae et al. 2021) and provided the first comprehensive view of the molecular ecology of Ni-CODHs. Our clustering-based strategy using both the MGnify and RefSeq/GenBank databases enabled the detection of phylogenetically diverse Ni-CODH sequences in half of all metagenome-derived assemblies from diverse biomes without any cultivation or primer bias (Figs. 1, S2, S3). Through the analyses, we proposed an additional Ni-CODH clade, clade H, and mini-CooS as a member of clade G. These data indicate that Ni-CODHs might be one of the prevalent enzymes across diverse microbiomes regardless of their substrate CO gas which is not prevalent on the Earth.

Our analysis revealed clade-specific distributions of Ni-CODHs in the biomes (Fig. 2). Especially, specific Ni-CODH clusters in clades B–E were identified mainly in the “host-associated” samples. The phylogenetic restriction of Ni-CODHs to the “host-associated” biomes is consistent with the phylogenetic niche conservatism at the protein level (Boyd et al. 2010; Pyron et al. 2015). A simple hypothesis was that taxonomic restriction in the abundant Ni-CODH owners might be occurred in the “host-associated” samples. In fact, the “host-associated” Ni-CODHs exhibited the low taxonomic diversity in which Firmicutes_A occupied ~ 75% of the Ni-CODH clusters (Fig. 3A) and the order Lachnospirales, major owner of the “host-associated” Ni-CODHs in Firmicutes_A is one of the most abundant taxa in human and mammals gut microbiomes (Meehan and Beiko 2014; Vacca et al. 2020). Thus, we speculated that highly biased microbial communities might have affected the composition of the gene pool and restricted the phylogenetic clades of Ni-CODHs in host-associated environments. However, the remaining 25% of the “host-associated” Ni-CODH clusters were distinct from Firmicutes_A at the phylum level (Fig. 3A) indicating that 10 distinct phyla possess phylogenetically closely related Ni-CODHs possibly through horizontal gene transfers. Although the organisms bearing the Ni-CODHs and their restricted habitats in the “host-associated” environments could be one of the main factors in determining the biased Ni-CODH distribution in microbiomes, horizontal gene transfers of Ni-CODHs within a particular environment might have also partly caused the observed restriction of Ni-CODH distributions in host-associated environments.

In contrast, the composition profiles of neighboring gene-associated functions did not show particular patterns but were similar among the three major biomes (Fig. 3B). Firmicutes_A and Desulfobacteota, Chloroflexota, Firmicutes_B, Halobacteriota and Thermoproteota, the major phyla of Ni-CODH owners in our analysis, contained various acetogens, methanogens, and carboxydotrophs to use the multiple Ni-CODHs for energy conservation and carbon fixation (Takors et al. 2018; Inoue et al. 2019a). Especially, in human gut microbiomes, the Ni-CODHs in the order Lachnospirales such as *Blautia hydrogenotrophica* are used for H_2_-dependent acetogenesis via WLP (Rey et al. 2010; Laverde Gomez et al. 2019). Moreover, luminal CO emission occurs via various microbial metabolic pathways as well as the host and carbon cycling is performed by CO-utilizing microbiomes (Hopper et al. 2020) where CO oxidation by Ni-CODH might be coupled with ECH or CooF/FNOR. Similarly, CO emission occurs via organic solid waste degradation processes (Haarstad et al. 2006; Stegenta-Dąbrowska et al. 2019), whereas most MAGs constructed in anaerobic digesters encode the Ni-CODH genes performing carbon cycle via methanogenesis and acetogenesis (Treu et al. 2018; Zhu et al. 2019; Campanaro et al. 2020). In natural aquatic environments, CO-mediated carbon cycle is performed by such carboxydotrophs (Conte et al. 2019); however, the direct involvement of Ni-CODH enzymes in the all the above CO-oxidation processes remains to be demonstrated. Our results representing phylogenetically diverse clades of Ni-CODHs prevalent in “environmental (aquatic)” suggested that Ni-CODHs might play an important ecological role even in aquatic environments containing less abundant CO.

## Supplementary Information

Below is the link to the electronic supplementary material.

Supplementary file1 (PDF 1154 KB)

Supplementary file2 (XLSX 728 KB)

## Abbreviations

Ni-CODH: Ni-containing carbon monoxide dehydrogenase
WLP: The Wood–Ljungdahl pathway
ECH: Energy converting hydrogenase
FNOR: Flavin adenine dinucleotide-dependent NAD(P) oxidoreductase
MAG: Metagenome-assembled genome
PCR: Polymerase chain reaction
NCBI: The National Centre for Biotechnology Information
COG: Cluster of orthologous groups

## References

[CR1] Adam PS, Borrel G, Gribaldo S (2018). Evolutionary history of carbon monoxide dehydrogenase/acetyl-CoA synthase, one of the oldest enzymatic complexes. Proc Natl Acad Sci USA.

[CR2] Benvenuti M, Meneghello M, Guendon C (2020). The two CO-dehydrogenases of *Thermococcus* sp. AM4. Biochim Biophys Acta Bioenerg.

[CR3] Boyd ES, Hamilton TL, Spear JR (2010). [FeFe]-hydrogenase in Yellowstone National Park: evidence for dispersal limitation and phylogenetic niche conservatism. ISME J.

[CR4] Buchfink B, Xie C, Huson DH (2014). Fast and sensitive protein alignment using DIAMOND. Nat Methods.

[CR5] Campanaro S, Treu L, Rodriguez-R LM (2020). New insights from the biogas microbiome by comprehensive genome-resolved metagenomics of nearly 1600 species originating from multiple anaerobic digesters. Biotechnol Biofuels.

[CR6] Can M, Armstrong FA, Ragsdale SW (2014). Structure, function, and mechanism of the nickel metalloenzymes, CO dehydrogenase, and acetyl-CoA synthase. Chem Rev.

[CR7] Capella-Gutiérrez S, Silla-Martínez JM, Gabaldón T (2009). trimAl: a tool for automated alignment trimming in large-scale phylogenetic analyses. Bioinformatics.

[CR8] Chaumeil PA, Mussig AJ, Hugenholtz P, Parks DH (2020). GTDB-Tk: a toolkit to classify genomes with the genome taxonomy database. Bioinformatics.

[CR9] Conte L, Szopa S, Séférian R, Bopp L (2019). The oceanic cycle of carbon monoxide and its emissions to the atmosphere. Biogeosciences.

[CR10] Dobbek H, Svetlitchnyi V, Gremer L (2001). Crystal structure of a carbon monoxide dehydrogenase reveals a [Ni-4Fe-5S] cluster. Science.

[CR11] Doukov TI, Iverson TM, Seravalli J (2002). A Ni-Fe-Cu center in a bifunctional carbon monoxide dehydrogenase/acetyl-CoA synthase. Science.

[CR12] Drennan CL, Heo J, Sintchak MD (2001). Life on carbon monoxide: X-ray structure of *Rhodospirillum rubrum* Ni-Fe-S carbon monoxide dehydrogenase. Proc Natl Acad Sci USA.

[CR13] Edgar RC (2010). Search and clustering orders of magnitude faster than BLAST. Bioinformatics.

[CR14] Evans PN, Parks DH, Chadwick GL (2015). Methane metabolism in the archaeal phylum Bathyarchaeota revealed by genome-centric metagenomics. Science.

[CR15] Fukuyama Y, Omae K, Yoshida T, Sako Y (2019). Transcriptome analysis of a thermophilic and hydrogenogenic carboxydotroph *Carboxydothermus pertinax*. Extremophiles.

[CR16] Fukuyama Y, Inoue M, Omae K (2020). Anaerobic and hydrogenogenic carbon monoxide-oxidizing prokaryotes: versatile microbial conversion of a toxic gas into an available energy. Adv Appl Microbiol.

[CR17] Galperin MY, Makarova KS, Wolf YI, Koonin EV (2015). Expanded microbial genome coverage and improved protein family annotation in the COG database. Nucleic Acids Res.

[CR18] Geelhoed JS, Henstra AM, Stams AJM (2016). Carboxydotrophic growth of *Geobacter sulfurreducens*. Appl Microbiol Biotechnol.

[CR19] Gong W, Hao B, Wei Z (2008). Structure of the α_2_ε_2_ Ni-dependent CO dehydrogenase component of the *Methanosarcina barkeri* acetyl-CoA decarbonylase/synthase complex. Proc Natl Acad Sci USA.

[CR20] Haarstad K, Bergersen O, Sørheim R (2006). Occurrence of carbon monoxide during organic waste degradation. J Air Waste Manag Assoc.

[CR21] Hoang DT, Chernomor O, von Haeseler A (2018). UFBoot2: improving the ultrafast bootstrap approximation. Mol Biol Evol.

[CR22] Hopper CP, De La Cruz LK, Lyles KV (2020). Role of carbon monoxide in host−gut microbiome communication. Chem Rev.

[CR23] Hoshino T, Inagaki F (2017). Distribution of anaerobic carbon monoxide dehydrogenase genes in deep subseafloor sediments. Lett Appl Microbiol.

[CR24] Inoue M, Nakamoto I, Omae K (2019). Structural and phylogenetic diversity of anaerobic carbon-monoxide dehydrogenases. Front Microbiol.

[CR25] Inoue M, Tanimura A, Ogami Y (2019). Draft genome sequence of *Parageobacillus thermoglucosidasius* strain TG4, a hydrogenogenic carboxydotrophic bacterium isolated from a marine sediment. Microbiol Resour Announc.

[CR26] Inoue M, Izumihara H, Fukuyama Y (2020). Carbon monoxide-dependent transcriptional changes in a thermophilic, carbon monoxide-utilizing, hydrogen-evolving bacterium *Calderihabitans maritimus* KKC1 revealed by transcriptomic analysis. Extremophiles.

[CR27] Inoue M, Tanimura A, Fukuyama Y (2020). Draft genome sequence of *Thermanaeromonas* sp. strain C210, isolated in the presence of carbon monoxide. Microbiol Resour Announc.

[CR28] Jain C, Rodriguez-R LM, Phillippy AM (2018). High throughput ANI analysis of 90K prokaryotic genomes reveals clear species boundaries. Nat Commun.

[CR29] Kalyaanamoorthy S, Minh BQ, Wong TKF (2017). ModelFinder: fast model selection for accurate phylogenetic estimates. Nat Methods.

[CR30] Katoh K, Standley DM (2013). MAFFT multiple sequence alignment software version 7: improvements in performance and usability. Mol Biol Evol.

[CR31] Laverde Gomez JA, Mukhopadhya I, Duncan SH (2019). Formate cross-feeding and cooperative metabolic interactions revealed by transcriptomics in co-cultures of acetogenic and amylolytic human colonic bacteria. Environ Microbiol.

[CR32] Liew F, Henstra AM, Winzer K (2016). Insights into CO_2_ fixation pathway of *Clostridium autoethanogenum* by targeted mutagenesis. mBio.

[CR33] Marchler-Bauer A, Bo Y, Han L (2017). CDD/SPARCLE: functional classification of proteins via subfamily domain architectures. Nucleic Acids Res.

[CR34] Matsen FA, Kodner RB, Armbrust EV (2010). pplacer: linear time maximum-likelihood and Bayesian phylogenetic placement of sequences onto a fixed reference tree. BMC Bioinform.

[CR35] Matson EG, Gora KG, Leadbetter JR (2011). Anaerobic carbon monoxide dehydrogenase diversity in the homoacetogenic hindgut microbial communities of lower termites and the wood roach. PLoS ONE.

[CR36] Meehan CJ, Beiko RG (2014). A phylogenomic view of ecological specialization in the Lachnospiraceae, a family of digestive tract-associated bacteria. Genome Biol Evol.

[CR37] Minh BQ, Schmidt HA, Chernomor O (2020). IQ-TREE 2: new models and efficient methods for phylogenetic inference in the genomic era. Mol Biol Evol.

[CR38] Mitchell AL, Almeida A, Beracochea M (2020). MGnify: the microbiome analysis resource in 2020. Nucleic Acids Res.

[CR39] Mu A, Thomas BC, Banfield JF, Moreau JW (2020). Subsurface carbon monoxide oxidation capacity revealed through genome-resolved metagenomics of a carboxydotroph. Environ Microbiol Rep.

[CR40] Nitschke W, Russell MJ (2013). Beating the acetyl coenzyme A-pathway to the origin of life. Philos Trans R Soc B Biol Sci.

[CR41] Oelgeschläger E, Rother M (2008). Carbon monoxide-dependent energy metabolism in anaerobic bacteria and archaea. Arch Microbiol.

[CR42] Omae K, Fukuyama Y, Yasuda H (2019). Diversity and distribution of thermophilic hydrogenogenic carboxydotrophs revealed by microbial community analysis in sediments from multiple hydrothermal environments in Japan. Arch Microbiol.

[CR43] Omae K, Oguro T, Inoue M (2021). Diversity analysis of thermophilic hydrogenogenic carboxydotrophs by carbon monoxide dehydrogenase amplicon sequencing using new primers. Extremophiles.

[CR44] Parks DH, Chuvochina M, Chaumeil PA (2020). A complete domain-to-species taxonomy for Bacteria and Archaea. Nat Biotechnol.

[CR45] Pyron RA, Costa GC, Patten MA, Burbrink FT (2015). Phylogenetic niche conservatism and the evolutionary basis of ecological speciation. Biol Rev.

[CR46] Rey FE, Faith JJ, Bain J (2010). Dissecting the in vivo metabolic potential of two human gut acetogens. J Biol Chem.

[CR47] Robb FT, Techtmann SM (2018). Life on the fringe: microbial adaptation to growth on carbon monoxide. F1000Research.

[CR48] Rother M, Oelgeschläger E, Metcalf WW (2007). Genetic and proteomic analyses of CO utilization by *Methanosarcina acetivorans*. Arch Microbiol.

[CR49] Sayers EW, Beck J, Brister JR (2020). Database resources of the National Center for Biotechnology Information. Nucleic Acids Res.

[CR50] Schoelmerich MC, Müller V (2019). Energy conservation by a hydrogenase-dependent chemiosmotic mechanism in an ancient metabolic pathway. Proc Natl Acad Sci USA.

[CR51] Schuchmann K, Müller V (2014). Autotrophy at the thermodynamic limit of life: a model for energy conservation in acetogenic bacteria. Nat Rev Microbiol.

[CR52] Singer SW, Hirst MB, Ludden PW (2006). CO-dependent H_2_ evolution by *Rhodospirillum rubrum*: role of CODH:CooF complex. Biochim Biophys Acta Bioenerg.

[CR53] Slobodkin A, Slobodkina G, Allioux M (2019). Genomic insights into the carbon and energy metabolism of a thermophilic deep-sea bacterium *Deferribacter autotrophicus* revealed new metabolic traits in the phylum *Deferribacteres*. Genes (basel).

[CR54] Soboh B, Linder D, Hedderich R (2002). Purification and catalytic properties of a CO-oxidizing:H_2_-evolving enzyme complex from *Carboxydothermus hydrogenoformans*. Eur J Biochem.

[CR55] Sokolova TG, Henstra AM, Sipma J (2009). Diversity and ecophysiological features of thermophilic carboxydotrophic anaerobes. FEMS Microbiol Ecol.

[CR56] Stegenta-Dąbrowska S, Drabczyński G, Sobieraj K (2019). The biotic and abiotic carbon monoxide formation during aerobic co-digestion of dairy cattle manure with green waste and sawdust. Front Bioeng Biotechnol.

[CR57] Takors R, Kopf M, Mampel J (2018). Using gas mixtures of CO, CO_2_ and H_2_ as microbial substrates: the do’s and don’ts of successful technology transfer from laboratory to production scale. Microb Biotechnol.

[CR58] Tan X, Loke HK, Fitch S, Lindahl PA (2005). The tunnel of acetyl-coenzyme A synthase/carbon monoxide dehydrogenase regulates delivery of CO to the active site. J Am Chem Soc.

[CR59] Techtmann SM, Lebedinsky AV, Colman AS (2012). Evidence for horizontal gene transfer of anaerobic carbon monoxide dehydrogenases. Front Microbiol.

[CR60] Treu L, Campanaro S, Kougias PG (2018). Hydrogen-fueled microbial pathways in biogas upgrading systems revealed by genome-centric metagenomics. Front Microbiol.

[CR61] Vacca M, Celano G, Calabrese FM (2020). The controversial role of human gut Lachnospiraceae. Microorganisms.

[CR62] Wareham LK, Southam HM, Poole RK (2018). Do nitric oxide, carbon monoxide and hydrogen sulfide really qualify as “gasotransmitters” in bacteria?. Biochem Soc Trans.

[CR63] Whitham JM, Tirado-Acevedo O, Chinn MS (2015). Metabolic response of *Clostridium ljungdahlii* to oxygen exposure. Appl Environ Microbiol.

[CR64] Wittenborn EC, Guendon C, Merrouch M (2020). The solvent-exposed Fe-S D-cluster contributes to oxygen-resistance in *Desulfovibrio vulgaris* Ni-Fe carbon monoxide dehydrogenase. ACS Catal.

[CR65] Yu G, Smith DK, Zhu H (2017). GGTREE: an R package for visualization and annotation of phylogenetic trees with their covariates and other associated data. Methods Ecol Evol.

[CR66] Zhu X, Campanaro S, Treu L (2019). Novel ecological insights and functional roles during anaerobic digestion of saccharides unveiled by genome-centric metagenomics. Water Res.

