# Supplementary material for: Biome-specific distribution of Ni-containing carbon monoxide dehydrogenases
Source: Extremophiles. 2022 Jan 20;26(1):9. doi: 10.1007/s00792-022-01259-y (PMC8776680; doi:10.1007/s00792-022-01259-y)
Supplement: Supplementary file 1 — Supplementary file1 (PDF 1154 KB) [file 792_2022_1259_MOESM1_ESM.pdf]

Supplementary Materials

Biome-Specific Distribution of Ni-Containing Carbon Monoxide Dehydrogenases

Masao Inoue[1,2,3,*], Kimiho Omae[4], Issei Nakamoto[1], Ryoma Kamikawa[1], Takashi Yoshida[1] and Yoshihiko Sako[1]

[1]Graduate School of Agriculture, Kyoto University, Kitashirakawa Oiwake-cho, Sakyo-ku, Kyoto, 606-8502, Japan

[2]R-GIRO, Ritsumeikan University, 1-1-1 Nojihigashi, Kusatsu, Shiga 525-8577, Japan

[3]College of Life Sciences, Ritsumeikan University, 1-1-1 Nojihigashi, Kusatsu, Shiga 525-8577, Japan

[4]Department of Integrated Biosciences, Graduate School of Frontier Science, The University of Tokyo, 5-1-5 Kashiwanoha, Kashiwa, Chiba 277-8561, Japan

*****Correspondence: mainoue@fc.ritsumei.ac.jp; Tel.: +81-77-599-4346

Supplementary Tables

Table S1. A dataset of 2,462 Ni-CODH clusters with biome categories, phylum-level taxonomies, and COGs for neighboring genes. Presented as a separate MS Excel file.

Supplementary Figures

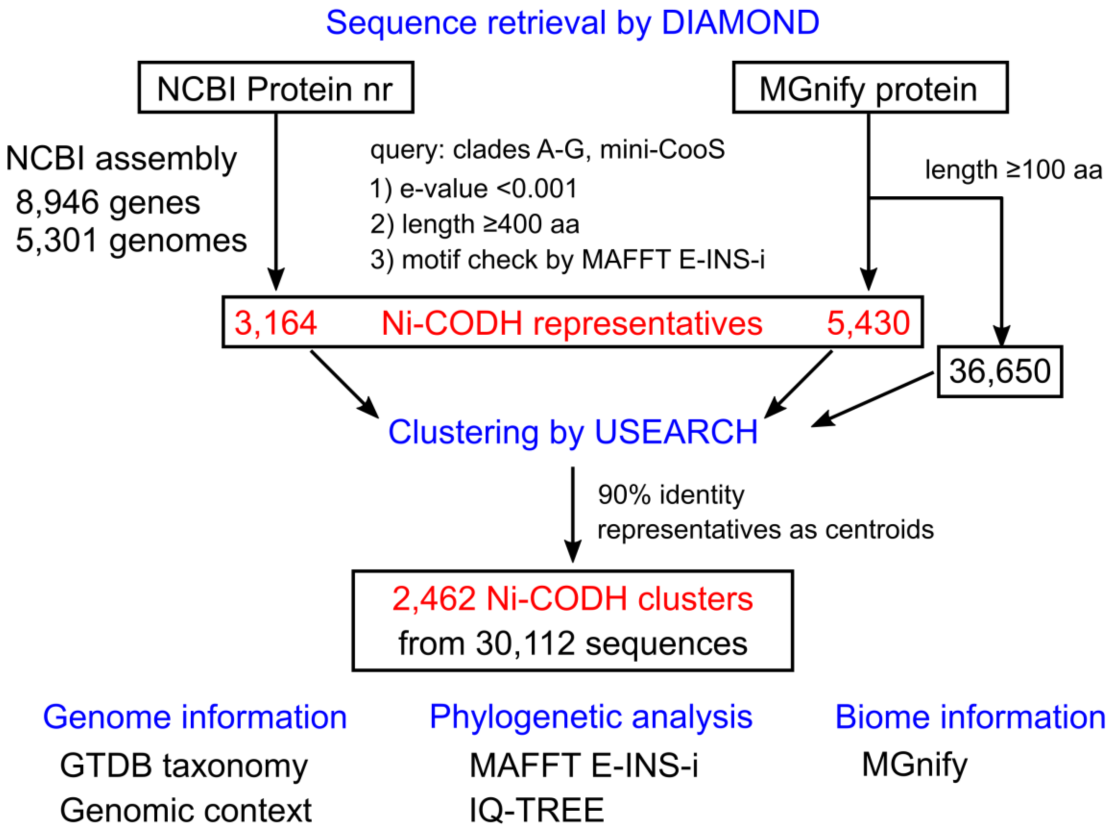

Figure S1. Pipelines for the data survey.

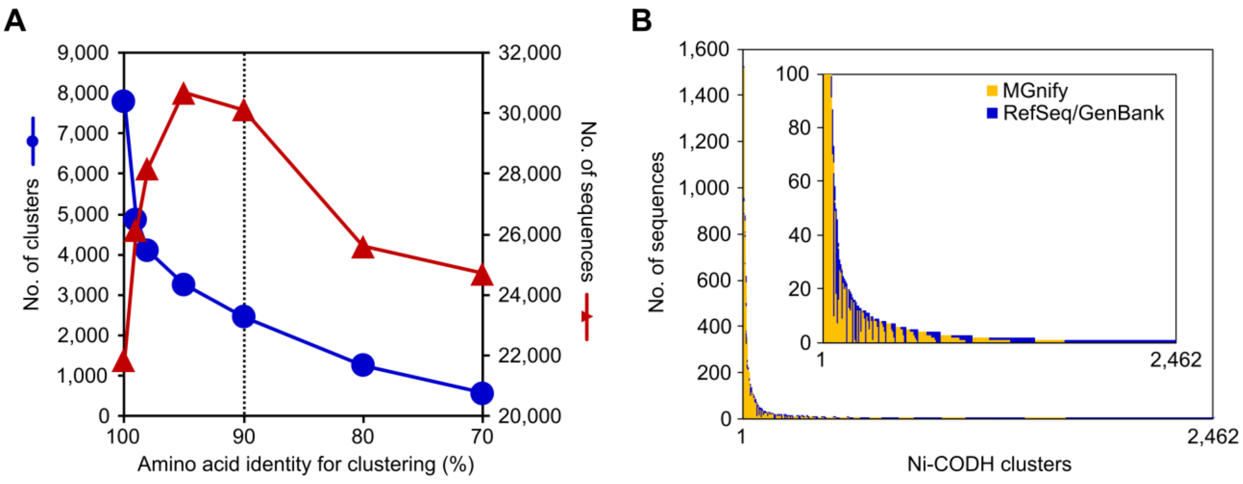

Figure S2. Clustering of Ni-CODH sequences. (**a**) The relationships between clustering criteria and efficiencies. (**b**) Frequency distribution of the numbers of Ni-CODH sequences from the MGnify and RefSeq/GenBank databases in each Ni-CODH cluster. *Inset*, enlarged view of the distribution.

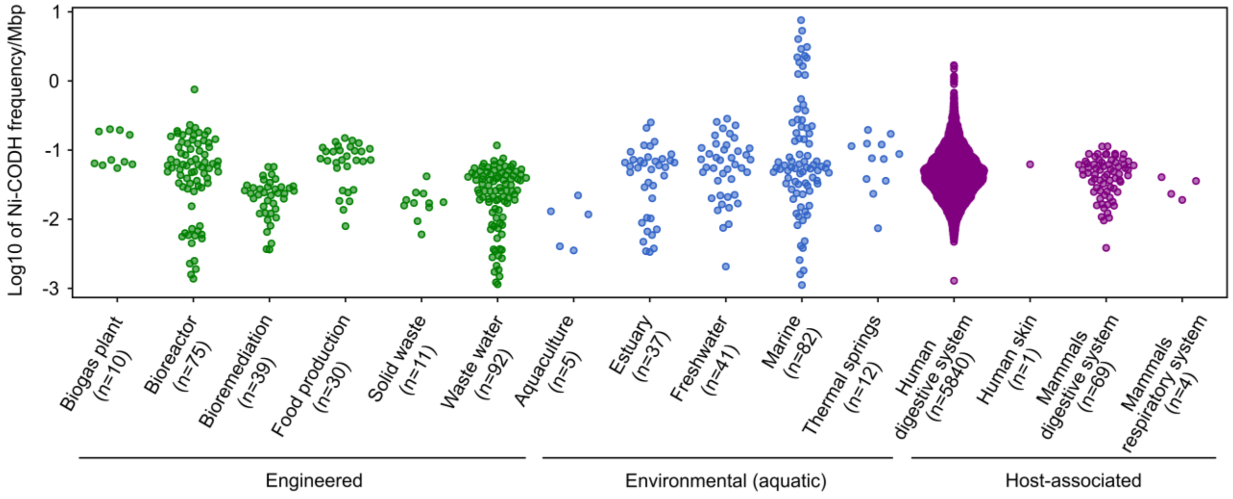

Figure S3. Appearance frequency of Ni-CODH sequences in 6,348 metagenome assemblies used for this study in each biome. The numbers of assemblies are indicated in parentheses.

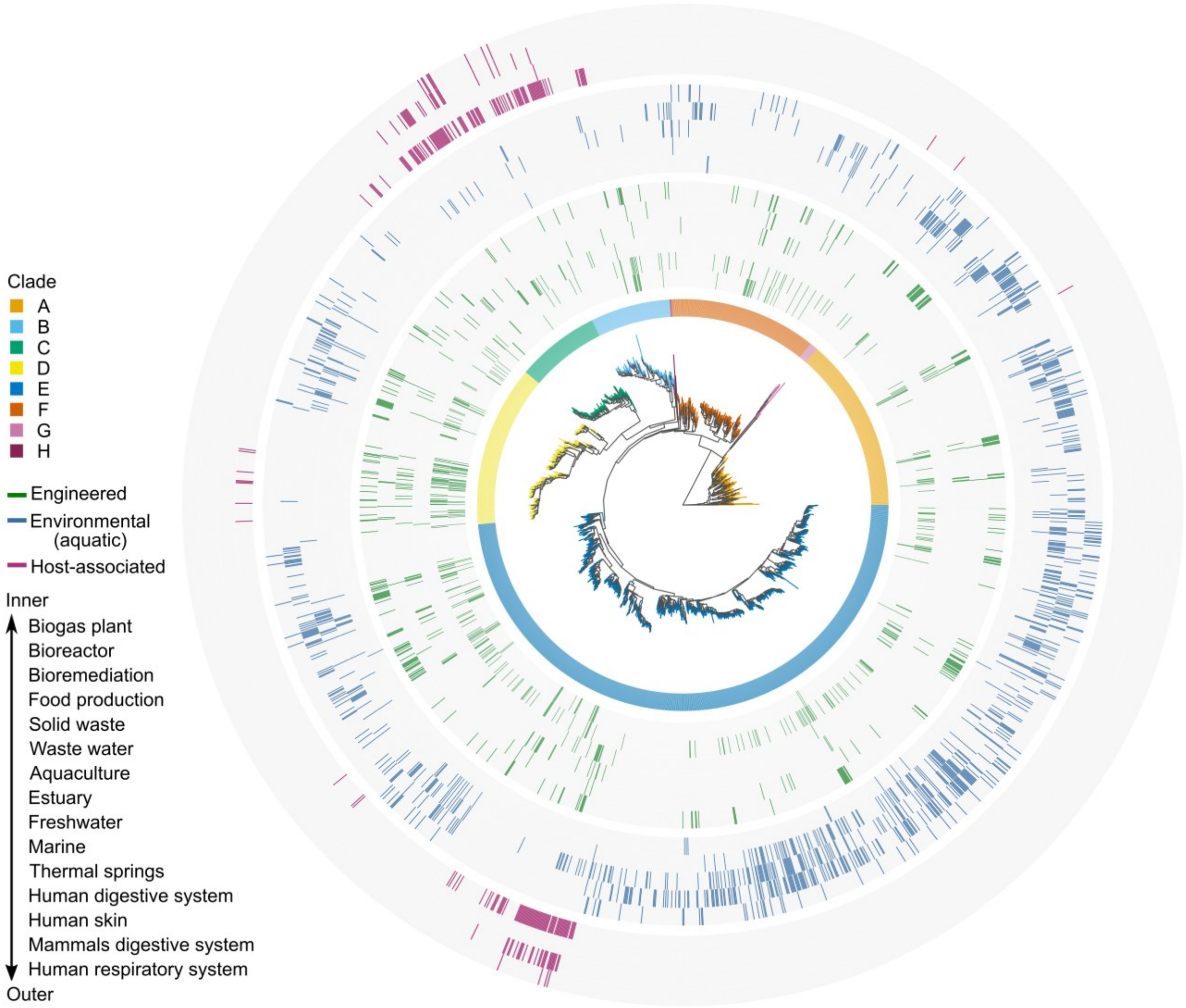

Figure S4. Mapping of biome subcategories to the whole Ni-CODH phylogeny.

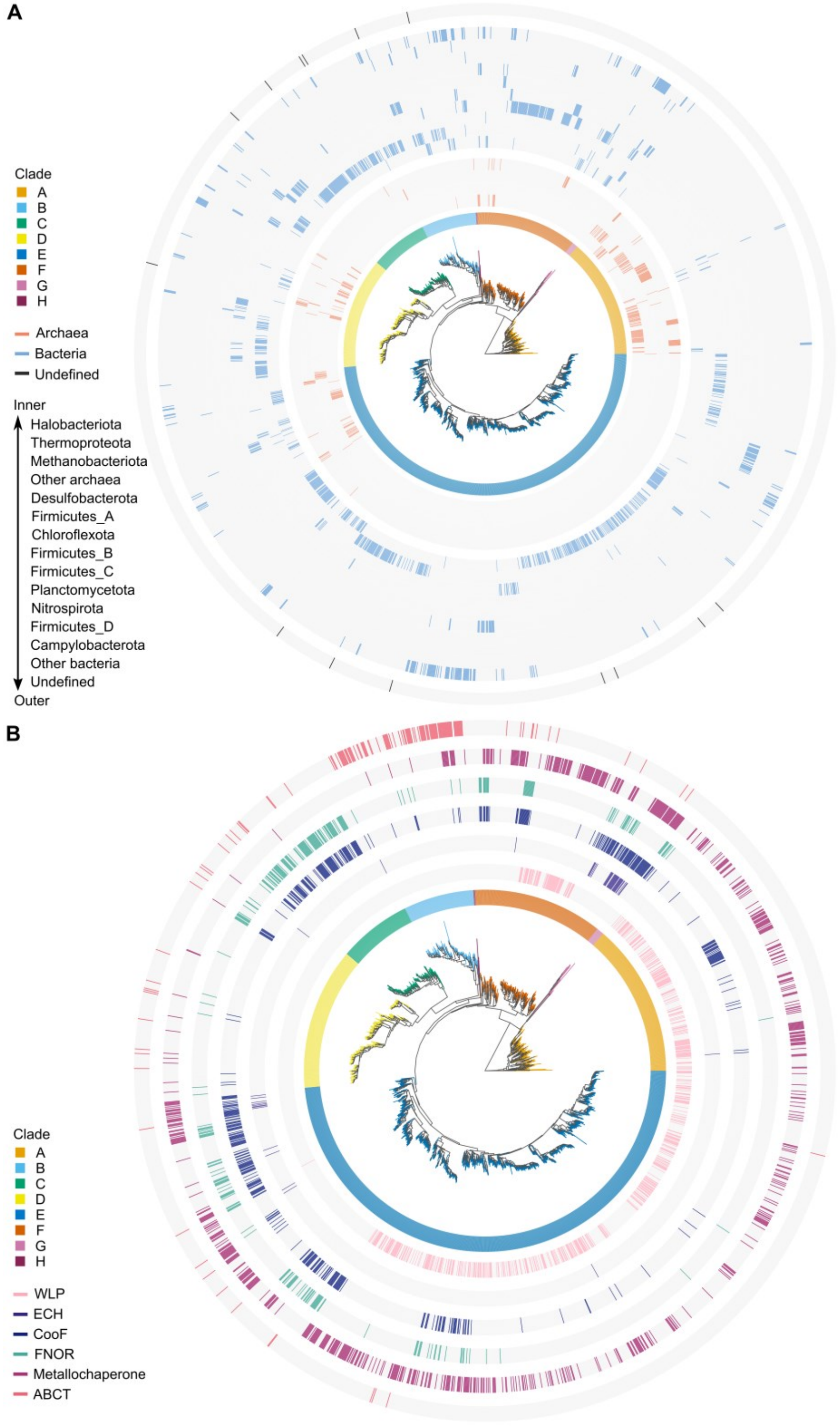

Figure S5. Mapping of taxonomy (A) and neighboring gene-associated function (B) to whole Ni-CODH phylogeny.
